# The cytosolic glyoxalases of *Plasmodium falciparum* are dispensable during asexual blood-stage development

**DOI:** 10.15698/mic2018.01.608

**Published:** 2017-11-20

**Authors:** Cletus A. Wezena, Romy Alisch, Alexandra Golzmann, Linda Liedgens, Verena Staudacher, Gabriele Pradel, Marcel Deponte

**Affiliations:** 1 Department of Parasitology, Ruprecht-Karls University, D-69120 Heidelberg, Germany.; 2 Division of Cellular and Applied Infection Biology, Institute of Zoology, RWTH Aachen University, D-52074 Aachen, Germany.; 3 Department of Chemistry/Biochemistry, University of Kaiserslautern, D-67663 Kaiserslautern, Germany.

**Keywords:** Plasmodium falciparum, malaria, glyoxalase, drug target, CRISPR/Cas9

## Abstract

The enzymes glyoxalase 1 and 2 (Glo1 and Glo2) are found in most eukaryotes and catalyze the glutathione-dependent conversion of 2-oxoaldehydes to 2-hydroxycarboxylic acids. Four glyoxalases are encoded in the genome of the malaria parasite *Plasmodium falciparum*, the cytosolic enzymes PfGlo1 and PfcGlo2, the apicoplast enzyme PftGlo2, and an inactive Glo1-like protein that also carries an apicoplast-targeting sequence. Inhibition or knockout of the *Plasmodium* glyoxalases was hypothesized to lead to an accumulation of 2-oxoaldehydes and advanced glycation end-products (AGE) in the host-parasite unit and to result in parasite death. Here, we generated clonal *P. falciparum *strain 3D7 knockout lines for *PFGLO1* and *PFcGLO2* using the CRISPR-Cas9 system. Although 3D7Δglo1 knockout clones had an increased susceptibility to external glyoxal, all 3D7Δglo1 and 3D7Δcglo2 knockout lines were viable and showed no significant growth phenotype under standard growth conditions. Furthermore, the lack of PfcGlo2, but not PfGlo1, increased gametocyte commitment in the knockout lines. In summary, PfGlo1 and PfcGlo2 are dispensable during asexual blood-stage development while the loss of PfcGlo2 may induce the formation of transmissible gametocytes. These combined data show that PfGlo1 and PfcGlo2 are most likely not suited as targets for selective drug development.

## INTRODUCTION

In 1990, Vander Jagt *et al*. reported that *Plasmodium falciparum*-infected erythrocytes do not only consume much more glucose than uninfected erythrocytes, but also produce up to 30-times more d-lactate owing to a highly efficient glyoxalase system 1. Since this seminal study, the *P. falciparum *glyoxalases have gained considerable attention as model enzymes 2,3,4,5 and as potential targets for rational drug development 6,7,8,9,10. The glyoxalase system, which usually consists of glutathione (or trypanothione in kinetoplastida), the isomerase glyoxalase 1 (Glo1) and the thioesterase glyoxalase 2 (Glo2), is found in most eukaryotes and several prokaryotes 5,11,12,13. The system is considered to be relevant for the detoxification of (glycolysis-derived) methylglyoxal and other 2-oxo-¬aldehydes, thus preventing the formation of advanced glycation end-products (AGE) with implications for diabetes, cancer and many other important diseases 14,15,16,17,18,19. However, 2-oxoaldehydes, glyoxalases and AGE can also have regulatory roles, and insular glyoxalases point to alternative functions 5,13,18,20,21,22,23,24,25.

The *P. falciparum* genome encodes four glyoxalases, two cytosolic and two apicoplast proteins 4,8,13. Cytosolic PfGlo1 and PfcGlo2 presumably constitute a functional glyoxalase system 4,7,8, whereas the Glo1-like protein PfGILP is inactive in standard enzyme assays and cannot provide canonical substrates for apicoplast PftGlo2 because of an altered active site 4,5,7,8. In addition, human erythrocytes harbour a functional hGlo1/hGlo2 couple 8,26,27. We previously characterized the effects of non-glutathione as well as glutathione-derived inhibitors on the two different active sites of recombinant PfGlo1 4,9. Two tight-binding Glo1 inhibitors and a variety of ester derivates were also characterized in cell culture experiments revealing micromolar IC_50_ values that were three to four orders of magnitude higher than the IC_50_ values with recombinant PfGlo1 9,10. Whether the glyoxalase system of the erythrocyte-*P. falciparum* host-parasite unit is indeed a suitable drug target remained to be shown. We therefore analyzed in the present study the relevance of the cytosolic *P. falciparum* glyoxalases for parasite survival using a combination of reverse genetics and biochemical assays. We found that PfGlo1 and PfcGlo2 are both dispensable for asexual blood-stage development while the loss of PfcGlo2 results in increased gametocyte commitment rates. Thus, PfGlo1 and PfcGlo2 are most likely not suited as targets for selective drug development and inhibition of PfcGlo2 might even promote the spread of malaria.

## RESULTS

### Generation and validation of glyoxalase knockout strains

**Figure 1:**
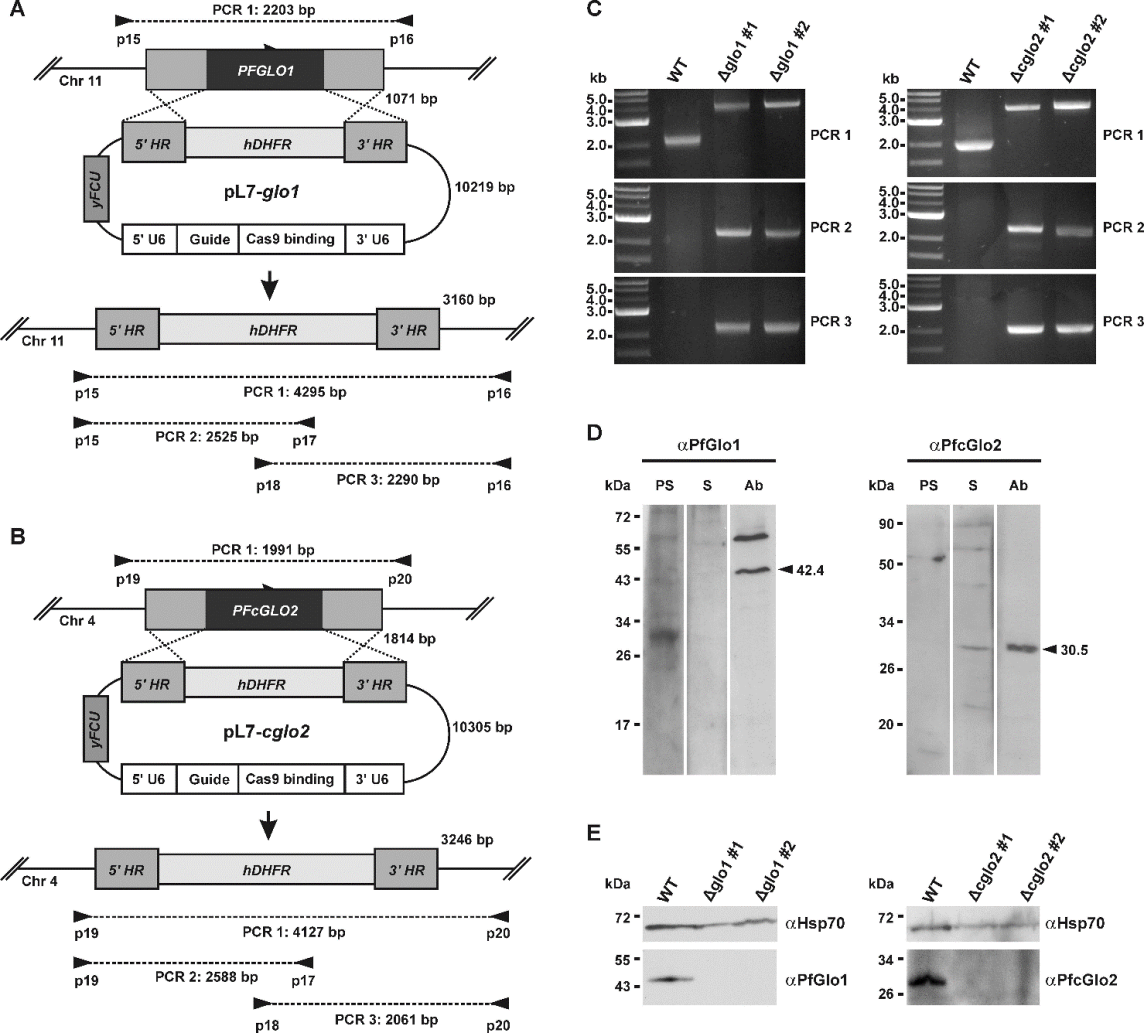
FIGURE 1: Generation and validation of *P. falciparum* 3D7Δglo1 and 3D7Δcglo2 knockout strains. **(A)** and **(B)** Knockout strategy for *PFGLO1* and *PFcGLO2* using the CRISPR-Cas9 system based on the method by Ghorbal *et al*. 29. Primer pairs and expected PCR products 1-3 for wild-type strain 3D7 and knockout parasite lines are indicated. **(C)** PCR controls with isolated genomic DNA from clonal parasite lines 3D7Δglo1 and 3D7Δcglo2 after transfection, selection and limited dilution. PCR products 1-3 are shown for two clones (#1 and #2) from each knockout experiment. Wild-type strain 3D7 served as a control. **(D)** Analysis of rabbit peptide antibodies against PfGlo1 and PfcGlo2. *P. falciparum* extracts from 2 x 10^7^ cells were loaded per lane on a 15% gel, separated by reducing SDS-PAGE and analyzed by western blotting. The expected sizes of PfGlo1 and PfcGlo2 are indicated by arrowheads. PS, S, Ab: Decoration with pre-immune serum, serum and affinity-purified antibody, respectively. **(E)** Western blot controls with extracts from wild-type strain 3D7 as well as two clonal 3D7Δglo1 and 3D7Δcglo2 parasite lines from panel C using the purified antibodies from panel D. Decoration with an antibody against Hsp70 served as a loading control.

Initial attempts to disrupt *PFGLO1* and *PFcGLO2* by double crossover recombination using the plasmid pHTK 28 were not successful despite a variety of selection protocols that combined positive selection with the dihydrofolate reductase inhibitor WR99210 and negative selection with the *Herpes simplex* thymidine kinase substrate ganciclovir (data not shown). We therefore speculated that both genes might be either essential or that the loci might be inaccessible to genetic manipulation 13. Following the establishment of the CRISPR-Cas9 system in *P. falciparum*, we were able to delete both genes using the method by Ghorbal *et al*. 29 (Fig. 1A, B). Successful deletion by double crossover recombination was validated for clonal 3D7Δglo1 and 3D7Δcglo2 parasite lines by analytical PCR (Fig. 1C). In addition, gene multiplications or other genetic events that might have resulted in functional PfGlo1 or PfcGlo2 were excluded by western blot analysis with specific antibodies (Fig. 1D), revealing that all clonal knockout strains lacked either PfGlo1 or PfcGlo2 as expected (Fig. 1E). In summary, *PFGLO1* and *PFcGLO2* are not essential for the asexual blood-stages of *P. falciparum*.

### Phenotypic analysis and enzyme activities of glyoxalase knockout strains 

**Figure 2:**
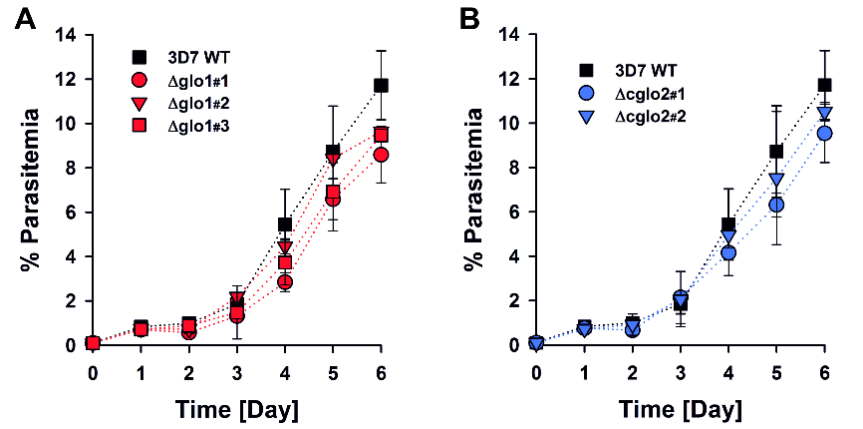
FIGURE 2: Growth curve analysis of *P. falciparum* knockout strains 3D7Δglo1 and 3D7Δcglo2. **(A)** Growth curves of three clonal 3D7Δglo1 parasite lines in comparison to wild-type strain 3D7. **(B)** Growth curves of two clonal 3D7Δcglo2 parasite lines in comparison with wild-type strain 3D7. All strains were diluted to an initial parasitemia of 0.1% and monitored by counting parasites in Giemsa-stained blood smears. All data points are the mean ± S.D. from three independent experiments. Statistical analysis using the one way ANOVA method in SigmaPlot 12.5 did not reveal a significant difference among strains (p > 0.05).

The phenotype of clonal 3D7Δglo1 and 3D7Δcglo2 parasite lines was analyzed for Giemsa-stained blood smears by light microscopy. None of the knockout strains had a suspicious morphology during asexual blood-stage development when compared to the wild-type line (Fig. S1). Even though growth rates appeared to be slightly reduced for single knockout clones, differences to the wild-type strain were not significant according to statistical analyses (Fig. 2).

**Figure 3:**
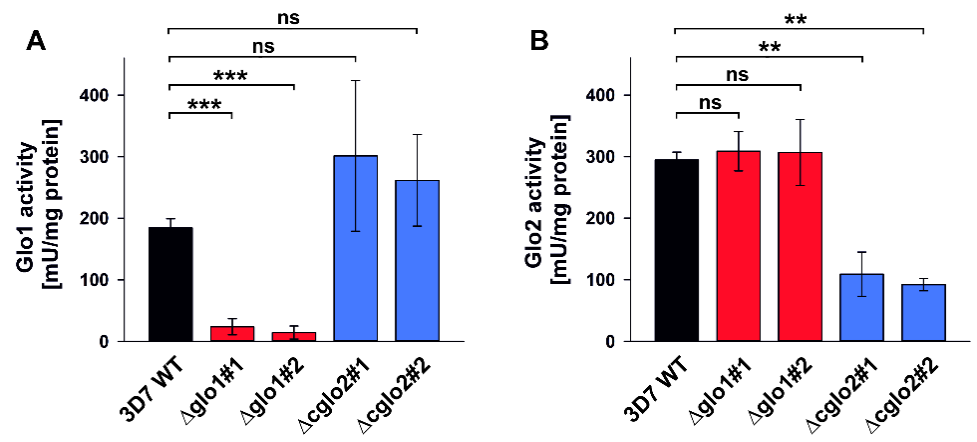
FIGURE 3: Glyoxalase activity measurements of *P. falciparum* knockout strains 3D7Δglo1 and 3D7Δcglo2 after erythrocyte removal by saponin lysis. **(A)** Normalized Glo1 activity measurements at 240 nm with parasite extracts from approximately 10^7^ trophozoites. **(B)** Normalized Glo2 activity measurements at 240 nm with parasite extracts from approximately 1.7 x 10^7^ trophozoites. All data points are the mean ± S.D. from two to four independent triplicate measurements. Statistical analyses were performed in SigmaPlot 12.5 using the one way ANOVA method (ns, not significant; ** p < 0.01; *** p < 0.001).

The contribution of PfGlo1 and PfcGlo2 to the overall Glo1 and Glo2 activities in purified parasite extracts was determined by comparing the 3D7 wild-type strain with clonal 3D7Δglo1 and 3D7Δcglo2 parasite lines (Fig. 3). The Glo1 activity of 3D7Δglo1 parasites dropped by approximately 90% (Fig. 3A), whereas 3D7Δglo2 parasites maintained about one third of the Glo2 activity (Fig. 3B). The latter result might be explained by the presence of (potentially dual localized) PftGlo2 in parasite extracts or the presence of human Glo2 that is taken up into the parasite. A residual Glo1 activity might either originate from a non-canonical parasite enzyme or from human Glo1 that is taken up. It is also worth mentioning that the Glo1 activity might be up-regulated in 3D7Δcglo2 parasites, even though averaged differences between strain 3D7 and the clonal knockout lines were not significant according to statistical analyses (Fig. 3A).

In summary, 3D7Δglo1 and 3D7Δcglo2 asexual blood-stage parasites have an inconspicuous morphology and no pronounced growth defect. The absent phenotype might be due to residual Glo1 and Glo2 activities that are detectable in purified knockout parasites. While residual Glo2 activities around 30 - 40% are plausible in the presence of PftGlo2, a residual Glo1 activity around 10% is quite surprising for 3D7Δglo1 knockout parasites considering the absence of another functional canonical Glo1-isoform in *P. falciparum*.

### Susceptibility of glyoxalase knockout strains to 2-oxoaldehydes

**Figure 4:**
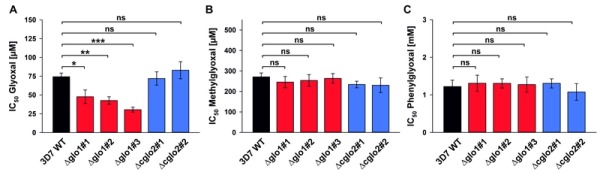
FIGURE 4: Growth inhibitory effects of exogenous 2-oxoaldehydes on synchronous ring-stage cell cultures of *P. falciparum* 3D7 wild-type strain and clonal 3D7Δglo1 and 3D7Δcglo2 knockout strains. IC_50_ values were determined in 96-well plates using a SYBR green 1 assay for bolus treatments with **(A)** glyoxal, **(B)** methylglyoxal and **(C)** phenylglyoxal after 72 h incubation. The dose response curves are shown in Fig. S2. All data points are the mean ± S.D. from technical triplicate measurements of three to seven independent experiments. Statistical analyses were performed in SigmaPlot 12.5 using the one way ANOVA method (ns, not significant; * p < 0.05; ** p < 0.01; *** p < 0.001).

Next, we analyzed the susceptibility of clonal 3D7Δglo1 and 3D7Δcglo2 parasite lines to external glyoxal, methylglyoxal and phenylglyoxal. Bolus treatments of ring-stage parasites revealed that the toxicity of the 2-oxoaldehydes depended on the size and/or polarity of the compounds with lowest IC_50_ values for glyoxal and highest IC_50_ values for phenylglyoxal (Fig. 4 and Fig. S2). The IC_50_ value for glyoxal decreased by up to 60% for the 3D7Δglo1 knockout parasites (Fig. 4A), whereas no significant differences between wild-type and 3D7Δglo1 knockout parasites were observed for methylglyoxal and phenylglyoxal (Fig. 4B, C). The susceptibility of the clonal 3D7Δcglo2 knockout parasites towards external 2-oxoaldehydes was similar to wild-type parasites (Fig. 4 and Fig. S2). In summary, 3D7Δglo1 knockout parasites have an increased susceptibility towards external glyoxal, which is more toxic for *P. falciparum* than methylglyoxal and phenylglyoxal.

### AGE accumulation in glyoxalase knockout strains

**Figure 5:**
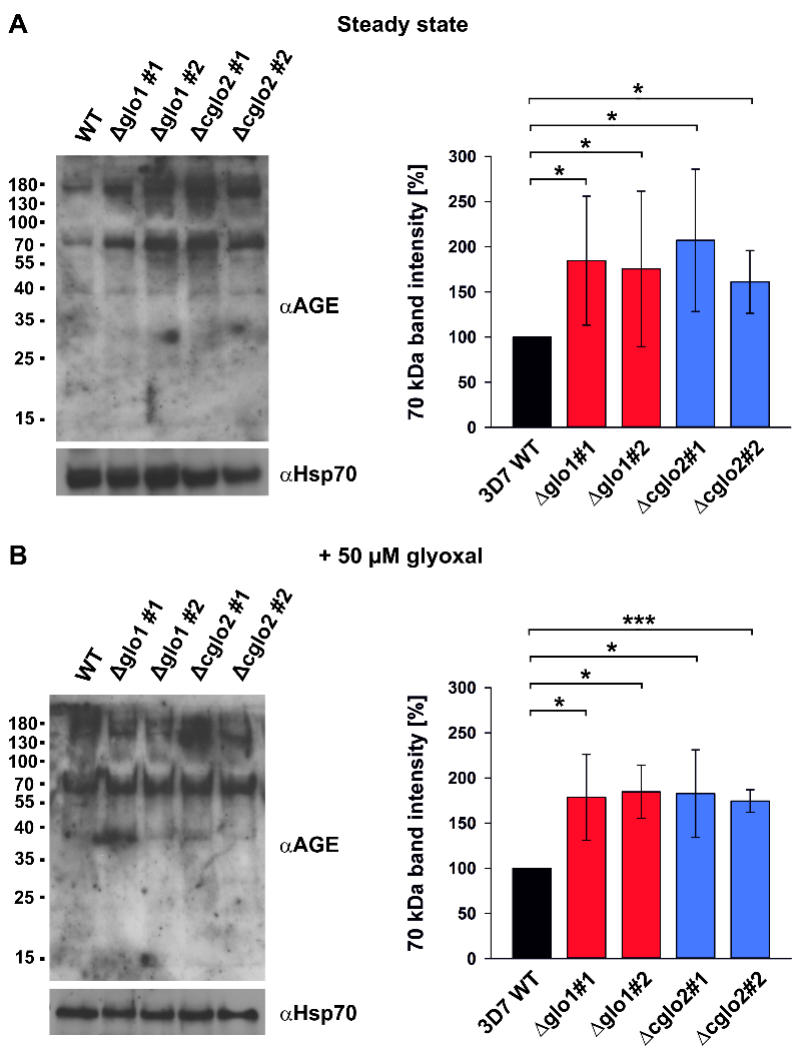
FIGURE 5: AGE detection by western blot analysis of parasite extracts from *P. falciparum* knockout strains 3D7Δglo1 and 3D7Δcglo2 after erythrocyte removal by saponin lysis. Wild-type parasites served as a control. **(A)** AGE detection under steady-state conditions without external 2-oxoaldehydes. **(B)** AGE detection after a 6 h bolus treatment with 50 µM glyoxal (which approximately corresponds to the IC_50_ values determined in Fig. S2). Trophozoite extracts from 2 x 10^7^ cells were loaded per lane on a 15% gel, separated by reducing SDS-PAGE and decorated with a commercial polyclonal anti-AGE antibody. Decoration against Hsp70 served as a loading control on a separate blot that was run in parallel using the same parasite extracts. Signal intensities were analyzed form three independent experiments using ImageJ and statistical analyses were performed in SigmaPlot 12.5 using the one way ANOVA method(* p < 0.05; *** p < 0.001).

**Figure 6:**
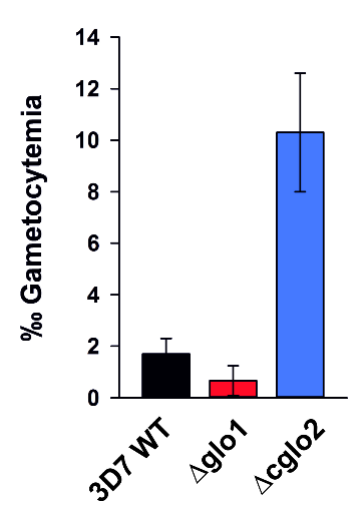
FIGURE 6: Gametocyte development of the *P. falciparum* knockout strains 3D7Δglo1 and 3D7Δcglo2. Gametocyte commitment was induced in 3D7Δglo1, 3D7Δcglo2 and wild-type 3D7 parasites and the gametocytemia was determined per 1000 red blood cells in Giemsa-stained blood smears 14 days post-induction. All data points are the mean ± S.D. from triplicate measurements. Results shown are representative of three independent experiments.

In order to address a potential AGE accumulation in glyoxalase knockout strains, we performed western blot analyses with purified trophozoite-stage parasite extracts using a commercial polyclonal anti-AGE antibody. Staining patterns with this antibody revealed a few bands and smears around 40, 70 and 180 kDa (Fig. 5). A general, unspecific accumulation of modified proteins was not detected. The staining patterns at 70 and 180 kDa were more intense for the 3D7Δglo1 and 3D7Δcglo2 parasite extracts than for wild-type parasite extracts. Band patterns were similar under steady-state conditions in the absence of external 2-oxoaldehydes (Fig. 5A) and 6 h after a bolus treatment with 50 µM glyoxal (Fig. 5B). In summary, western blot analyses support the accumulation of selected modified proteins in glyoxalase knockout strains. However, the growth of asexual blood-stage parasites appears to be unaffected by these protein modifications.

### Gametocytogenesis of glyoxalase knockout strains

In a final set of experiments, we aimed to investigate how the loss of PfGlo1 or PfcGlo2 activity as well as the accumulation of AGE would affect gametocyte commitment, thus the ability of the *P. falciparum* blood stages to enter the sexual pathway and to form transmissible gametocytes. Gametocyte cultures were set up for the clonal 3D7Δglo1 and 3D7Δcglo2 parasite lines as well as the parental wild-type 3D7 line used as control. Notably, the parental 3D7 line in general was a low gametocyte producer compared to standard gametocyte-producing strains such as NF54, because 3D7 has not been selected in the past for the ability to develop gametocytes. Parasite lines 3D7Δglo1 and 3D7Δcglo2 were both able to undergo gametocyte commitment and to develop mature gametocytes, and the gametocyte stages formed during maturation exhibited morphologies comparable to the wild-type strain (Fig. S1). Compared to its parental line, 3D7Δglo1 parasites formed reduced numbers of gametocytes in more than three independent experiments, when these were quantified 14 days after setting up the gametocyte cultures. It was noteworthy, though, that 3D7Δcglo2 parasites had an increased gametocytemia compared to the parental line in all of the experiments (Fig. 6), indicating that the lack of PfcGlo2 results in increased gametocyte induction.

## DISCUSSION

The cytosolic *P. falciparum *glyoxalases are dispensable during asexual blood-stage development in contrast to previous analysis using pHTK vectors 13, and glyoxalase knockout parasites have no significant growth defect. Our study exemplifies that negative results from traditional knockout attempts in *P. falciparum *are difficult to interpret because of methodological limitations, even when positive and negative selections are applied 28. Some of these limitations can be overcome by the targeted introduction of DNA double-strand breaks with the help of the CRISPR-Cas9 system 29. Nevertheless, also the CRISPR-Cas9 system has limitations and four attempts of our laboratory to generate 3D7Δtglo2 and 3D7Δgilp knockout parasites (PlasmoDB annotations PF3D7_1205700 and PF3D7_0604700) using pL7 plasmids have failed to date (data not shown). We consider the selection of the guide RNA or of the extremely AT-rich homology regions for *PFtGLO2* and *PFGILP *as likely methodological causes for the negative outcome of these knockout attempts rather than essential roles of both genes. This interpretation is also supported by a recent study in the rodent parasite *P. berghei*, revealing that the homologs *PBtGLO2* (PBANKA_0604400) and *PBGLO1 *(PBANKA_0933900) are dispensable and that knockout parasites have no growth defect 30 (the genes *PBcGLO2* (PBANKA_1004000) and *PBGILP* (PBANKA_0103400) were not covered in the genetic screen).

The dispensability of *PFGLO1* for parasite survival also explains the high IC_50_ values of tight-binding Glo1-inhibitors in previous cell culture experiments 10 in contrast to low nanomolar inhibition constants against recombinant PfGlo1 9. In accordance with these results, a single IC_50 _experiment with 3D7Δglo1 and 3D7Δcglo2 knockout strains and the Glo1 inhibitors compound 3 and 7 of our previous study 10 showed very similar IC_50_ values compared to wild-type parasites (data not shown). If human Glo1 compensated for the loss of PfGlo1 in the 3D7Δglo1 knockout strains, one would expect altered IC_50_ values because the inhibitors can also slowly inactivate the human enzyme 10. Thus, parasite killing of wild-type and knockout strains at micromolar instead of nanomolar Glo1 inhibitor concentrations can be attributed to off-target effects. Regarding the dispensability of *PFcGLO2* for parasite survival, it might be necessary to also delete *PFtGLO2* and/or to inactivate human Glo2 to cause a growth defect. However, this means that an inhibitor would have to target not only one but two or even three enzymes at once. Altogether our data suggest that PfGlo1 and PfcGlo2 are most likely not suited as targets for selective drug development.

One significant difference between wild-type and glyoxalase knockout parasites was observed for 3D7Δglo1 parasites that were treated with external glyoxal, whereas methylglyoxal or phenylglyoxal had similar effects on wild-type and knockout parasites. The results might be explained by different kinetics for the transport of glyoxal, methylglyoxal and phenylglyoxal across the three membranes that surround the parasite cytosol or by different glyoxalase efficiencies of the host-parasite unit regarding the conversion of these 2-oxoaldehydes. A third explanation could be that other enzymes of the host-parasite unit, such as 2-oxoaldehyde reductases or dehydrogenases, are able to metabolize methylglyoxal and phenylglyoxal but not glyoxal with sufficient efficiencies in the knockout strains.

An interesting observation was that the loss of PfcGlo2 in the asexual blood stages resulted in increased gametocyte formation. Commitment to gametocyte development is believed to take place at some point before schizogony, when individual schizonts produce merozoites that develop into gametocytes after invasion of red blood cells 31,32. A number of recent studies have demonstrated that the switch between asexual blood stage replication and gametocyte formation in *P. falciparum* is epigenetically regulated, involving *P. falciparum* heterochromatin protein 1 and histone deacetylase 2, as well as down-stream transcription factors such as Ap2G 33,34,35,36. Gametocyte commitment is assigned to a variety of internal and external stress factors such as high parasitemia, anemia, host immune response or drug treatment, but also endoplasmic reticulum stress 36,37,38,39,40,41,42,43. AGE accumulation appeared to be similar in 3D7Δglo1 and 3D7Δcglo2 knockout parasites and the lack of functional PfGlo1 did not trigger gametocyte commitment. We therefore speculate that the accumulation of *S*-d-lactoylglutathione or another thioester substrate of PfcGlo2 1,3,8 might have caused the induction of gametocyte commitment. Altered *S*-d-lactoylglutathione concentrations were previously reported to activate *Escherichia coli* potassium channels that share properties with cation-translocating channels in eukaryotes 20,24,44. Furthermore, altered *S*-d-lactoylglutathione concentrations might also affect the uptake of glutathione into organelles as previously reported for isolated rat liver mitochondria 25. Whether similar *S*-d-lactoylglutathione-dependent mechanisms play a role in gametocyte commitment remains to be studied.

## MATERIALS AND METHODS

### Materials

The Cas9-encoding plasmid pUF1-Cas9 and plasmid pL6, containing the single guide RNA (sgRNA) with a Cas9-binding sequence, were generously provided by Jose-Juan Lopez-Rubio 29. Cloning vector pCRII-TOPO was from Life technologies. Primers were from Metabion and *Taq* DNA polymerase and restriction enzymes were from New England Biolabs. The In-Fusion HD cloning kit was from Clontech. RPMI-1640 medium and albumax II were from Gibco Life Technologies. Hypoxanthine and gentamicin were from C.C.Pro GmbH or Gibco Life Technologies. Peptides and rabbit peptide antibodies against PfGlo1 (NH_2_-CLKYQTDEDYENFKQSWEPV-CONH_2_) and PfcGlo2 (NH_2_-CSAYEPTPGVNEKVYDGQ-CONH_2_) were generated by Pineda Antibody Service. SulfoLink resin was from Pierce. The polyclonal anti-AGE antibody (ab23722) was from Abcam and the goat anti-Rabbit IgG (H+L)-HRP conjugate was from BioRad. Saponin, blotting-grade milk powder, glyoxal, methylglyoxal and phenylglyoxal were from Sigma.

### Cloning of pL7 constructs

The pL7 transfection plasmids containing the single guide RNA (sgRNA) expression-cassette and the 5’- and 3’-homology regions of *PFGLO1* and *PFcGLO2* (PlasmoDB annotations PF3D7_1113700 and PF3D7_0406400) were generated from plasmid pL6 by multiple cloning steps. First, to generate plasmid pL6-*PFGLO1*, the 5’- and 3’-homology regions of *PFGLO1 *were PCR-amplified from *P. falciparum* strain 3D7 genomic DNA using primer sets P1/P2 and P3/P4, respectively (Table S1). PCR products were initially cloned into pCRII-TOPO. The 5’- and 3’-homology regions were subsequently excised from the TOPO vector, using *Eco*RI/*Nco*I for the 3’-fragment and *Sac*II/*Xba*I for the 5’-fragment, and cloned into pL6, using the restriction sites *Eco*RI/*Nco*I for the 3’-fragment and *Sac*II/*Spe*I for the 5’-fragment. Plasmid pL6-*PFcGLO2* was cloned analogously using primers set P5/P6 and P7/P8 (Table S1). Plasmids pL7-*PFGLO1* and pL7-*PFcGLO2* were subsequently generated by replacing the *Btg*ZI-adaptor of pL6-*PFGLO1* and pL6-*PFcGLO2* with the guide RNA (gRNA) fragment using the In-Fusion HD cloning kit. Briefly, the pL6 plasmids were digested stepwise with *Avr*II and *Btg*ZI for 2 h at 37°C and 2 h at 60°C, respectively. The 20-base gRNA fragments for each target were designed using the Protospacer software, flanked with 15 bases for In-Fusion cloning and purchased as complementary oligonucleotides P9/P10 and P11/P12 for pL7-*PFGLO1* and pL7-*PFcGLO2*, respectively (Table S1). The complementary oligonucleotides were mixed, annealed and ligated with the purified linearized pL6 plasmids in the In-fusion reaction. Stellar chemical competent cells were subsequently transformed with the In-Fusion reaction products. Correct sequences for the 5’- and 3’-homology regions as well as the sgRNA cassettes of pL7-*PFGLO1* and pL7-*PFcGLO2 *were confirmed by commercial DNA sequencing at GATC Biotech.

### Parasite culture and transfections

Asexual *P. falciparum *blood**-**stage parasites were cultured in fresh human A+ erythrocytes according to Trager and Jensen 45 at 36.5°C, 5% O_2_, 5% CO_2_ and 80% humidity in RPMI-1640 medium supplemented with 0.45% albumax II, 0.2 mM hypoxanthine and 2.7 µg/ml gentamicin. The hematocrit was 3.5% and the standard culture volume was 14 ml. Synchronised parasite cultures were obtained after treatment with 5% sorbitol 46. Transfections were conducted by electroporation (pre-loading) of uninfected erythrocytes with 100 µg pUF1-Cas9 or pL7 at 0.3 kV and 950 µF followed by the infection with *P. falciparum *3D7 parasites 47,48. Parasites were first transfected with plasmid pUF1-Cas9 and selected with 100 nM atovaquone followed by transfection with plasmid pL7-*PFGLO1* or pL7-*PFcGLO2* and selection with 2.7 nM WR99210. The drug pressure was applied 20 h post-transfection. For the first 7 days, drug and medium were renewed every 24 h. Thereafter, drug and medium were renewed every other day, and 70 µl of fresh erythrocytes were added to the culture once every week until parasites were detected in Giemsa-stained blood smears. A negative selection against parasites with non-integrated pL7 plasmids was conducted with 40 µM 5-flourocytosine. Clonal cell lines were obtained by limited dilution in 96-well plates.

### PCR analysis of parasite strains

The uptake of pUF1-Cas9 and the targeted disruption of the glyoxalases by the insertion of the dihydrofolate reductase gene were verified by PCR analysis using primer pairs P13/P14 for pUF1-Cas9, P15/P16, P15/P17, and P18/P16 for the disruption of *PFGLO1* and P19/P20, P19/P17, and P18/P20 for the disruption of *PFcGLO2 *(Table S1). Genomic DNA for PCR analysis was isolated with phenol/chloroform from late schizont/early ring-stage parasites at approximately 8% parasitemia as described previously 49.

### Purification of peptide antibodies 

Rabbit peptide antibodies against PfGlo1 and PfcGlo2 were purified from sera of immunized animals by affinity chromato-graphy. In brief, cysteine-containing peptides (1 mg/ml) were reduced with 25 mM borohydride in 50 mM Tris/HCl, 5 mM EDTA, pH 8.5 at 4°C overnight. The reduced peptides were coupled for 45 min to 1 ml SulfoLink resin. Unspecific binding sites were subsequently blocked with 50 mM cysteine in 50 mM Tris, 5 mM EDTA, pH 8.5. Peptide antibodies were affinity-purified from rabbit sera according to the manufacturer’s protocol. Antibody-containing eluate fractions were identified after SDS-polyacrylamide gel electrophoresis (SDS-PAGE) on Coomassie-stained 15% gels.

### Western blot analysis

Trophozoite extracts were prepared by saponin lysis 50,51 from 35 ml cultures with 5% parasitemia. Briefly, after centri-fugation at room temperature for 5 minutes at 755 *g*, erythrocytes were resuspended in ice-cold phosphate buffered saline (PBS) containing 0.05% (w/v) saponin and incubated for 60 sec on ice. The erythrocyte lysate was centrifuged at 4°C for 10 min at 1800 *g*. The supernatant was discarded and the parasite pellet was washed twice with PBS and once with PBS containing Roche complete protease inhibitor (PBS/PI). Purified parasites were resuspended in 50 µl of PBS/PI and disrupted by four freeze-thaw cycles comprising 5 min freezing in liquid nitrogen, 1 min thawing in a 37°C water bath and 30 sec of mixing on a vortex. The parasite extract was supplemented with 50 µl of 2 x Laemmli buffer containing 30% (v/v) 2-mercapto-ethanol and boiled at 95°C for 5 min. Protein extracts of approximately 2 x 10^7 ^purified trophozoites per lane were separated by SDS-PAGE and subsequently transferred to a nitrocellulose membrane by semi-dry blotting. The membrane was blocked for 1 h at room temperature with 5% (w/v) milk powder in 0.9% (w/v) NaCl, 10 mM Tris/HCl, pH 7.4 (milk/TBS) and incubated with preimmune serum, serum or purified antibodies in milk/TBS at 4°C overnight. For the detection of PfGlo1, PfcGlo2 and AGE, the purified peptide antibodies αPfGlo1 and αPfcGlo2 as well as the commercial anti-AGE antibody were diluted 1:500, 1:1000 and 1:500, respectively. The secondary goat anti-Rabbit antibody was diluted 1:10000 in 5% milk/TBS. At least three biological replicates were performed for each western blot analysis. Signal intensities were analyzed with ImageJ 52.

### Growth curve analysis

The growth phenotype of three clonal 3D7Δglo1 and two clonal 3D7Δcglo2 knockout strains was analysed in comparison with the 3D7 wild-type strain. Asynchronous standard cultures of each strain were adjusted to a starting parasitemia of 0.1% and maintained for six days. The medium was changed once every 24 h for the first three days and then twice every 24 h. Parasite morphology and parasitemia of each culture were daily assessed by light microscopy. Approximately 750 - 1500 erythrocytes were analyzed per Giemsa-stained blood smear. The average parasitemia was calculated for each time point from three independent biological replicates. Statistical analysis was performed in SigmaPlot 12.5 using the one way ANOVA method. Growth inhibition of synchronous ring-stage wild-type as well as 3D7Δglo1 and 3D7Δcglo2 knockout parasites by exogenous glyoxal, methylglyoxal or phenylglyoxal was determined in 96-well plates using a SYBR green 1 assay as described previously 53,54. Briefly, 72 h after the bolus treatments, the SYBR green fluorescence was measured in a microplate reader (BMG Latech, Germany), and IC_50_ values were computed from sigmoidal dose-response curves using the four parameter Hill function in SigmaPlot 12.5.

### Gametocyte formation assay 

Wild-type as well as 3D7Δglo1 and 3D7Δcglo2 knockout parasites were cultured as described above except for the replacement of albumax with 10% inactivated A+ serum as described previously 55. Two days after synchronization with 5% sorbitol, gametocyte commitment was induced by setting up the culture at 2% parasitemia and a haematocrit of 10% in 5 ml of the human A+ medium. The medium was replaced daily and Giemsa-stained blood smears were analyzed 14 d post-induction of gametocyte commitment. The gametocytemia was determined per 1000 red blood cells and the gametocyte stages II-V were counted in triplicate. Data analysis was performed using MS Excel 2010 and GraphPad Prism 5.

### Glyoxalase activity assays

Parasite lysates for glyoxalase activity measurements were prepared from 35 ml cultures of synchronous trophozoite-stage parasites using a slightly modified lysis protocol 7. Briefly, infected erythrocytes were first pelleted and then resuspended and lysed in 20 volumes of modified PBS containing 7 mM K_2_HPO_4_, 1 mM NaH_2_PO_4_, 11 mM NaHCO_3_, 58 mM KCl, 56 mM NaCl, 1 mM MgCl_2_, 14 mM glucose and 0.02% (w/v) saponin (pH 7.5 at 25°C). After incubation for 10 min at 37°C, erythrocyte lysates were centrifuged at 25°C for 3 min at 1500 *g* and the pellet was washed three times with modified PBS. About 5 x 10^7 ^purified trophozoites were resuspended in 150 μl of modified PBS and disrupted by four freeze-thaw cycles. The parasite lysate was centrifuged at 4°C for 60 min at 20800 *g* and the supernatant was transferred to a fresh precooled reaction tube and stored on ice for immediate Glo1 and Glo2 activity assays. The protein content of the parasite lysate supernatant was subsequently determined in a Bradford assay at 595 nm with bovine serum albumin as a standard 56.

Glo1 activities form parasite extracts were determined at 25°C using a thermostatted Jasco V-550 UV-vis spectrophotometer by following the formation of *S*-d-lactoylglutathione and the consumption of the hemithioacetal between methylglyoxal and reduced glutathione (GSH) at 240 nm using an extinction coefficient of ε_240nm _= 2.86 mM^−1^cm^−1^ 10,57. Briefly, stock solutions of 100 mM GSH and 100 mM methylglyoxal were freshly prepared in water and stored on ice. The hemithioacetal substrate was formed by mixing 100 µl of pre-warmed 1 M K_x_H_y_PO_4_, pH 7.0 and 100 µl 1 M KCl with 10 µl of 100 mM GSH and 20 µl of 100 mM methylglyoxal in 740 µl of pre-warmed double distilled water in a 1 ml cuvette. The mixture was incubated for 15 min at 25°C. After 14 min incubation, the measurement was set to ’auto zero’, and a baseline was recorded after 14.5 min for 30 sec before adding 30 µl of parasite lysate supernatant from approximately 10^7^ trophozoites to the mixture. After thorough mixing, the change in absorbance at *A*_240_ was immediately monitored. The slope of the initial increase of absorbance over time (d*A*_240_/d*t*)_0 _was determined and the activity of Glo1 (a_Glo1_) was calculated using a_Glo1 _= (d*A*_240_/d*t*)_0_/2.86 [mM/min].

Glo2 activities were measured analogously at 25°C by following the consumption of *S*-d-lactoylglutathione at 240 nm using an extinction coefficient of ε_240nm _= 3.1 mM^−1^cm^−1^ 10,57. Briefly, 100 mM Tris/HCl, pH 7.4 at 25°C and 3 mM *S*-d-lactoylglutathione in Tris buffer were freshly prepared and stored on ice. The assay setup comprised mixing 500 µl of pre-warmed Tris buffer, 100 µl of 3 mM *S*-d-lactoylglutathione and 350 µl of pre-warmed water in a 1 ml cuvette at 25°C. The measurement was set to ‘auto zero’ and a baseline was recorded for 30 seconds before adding 50 µl of parasite lysate supernatant from approximately 1.7 x 10^7^ trophozoites to the mixture. After thorough mixing, the change in absorbance at *A*_240_ was immediately monitored. The slope of the initial decrease of absorbance over time (d*A*_240_/d*t*)_0 _was determined and the activity of Glo2 (a_Glo2_) was calculated using a_Glo2_ = -(d*A*_240_/d*t*)_0_/3.1 [mM/min].

Glo1 and Glo2 activities were corrected for the dilution factor of the parasite lysate and normalized to the protein content of the lysate (yielding mU/mg_ (total protein)_ with 1 mU being the amount of enzyme that converts 1 nmol of substrate per min). The normalized Glo1 and Glo2 activities were averaged from two to four independent triplicate measurements. A statistical analysis was performed in SigmaPlot 12.5 using the one way ANOVA method.

## SUPPLEMENTAL MATERIAL

Click here for supplemental data file.

All supplemental data for this article are also available online at http://microbialcell.com/researcharticles/alcohols-enhance-the-rate-of-acetic-acid-diffusion-in-s-cerevisiae-biophysical-mechanisms-and-implications-for-acetic-acid-tolerance/.
