# Supplementary material for: The cytosolic glyoxalases of Plasmodium falciparum are dispensable during asexual blood-stage development
Source: Microb Cell. 2017 Nov 20;5(1):32–41. doi: 10.15698/mic2018.01.608 (PMC5772037; doi:10.15698/mic2018.01.608)
Supplement: Supplementary file 1 [file mic-05-032-s01.pdf]

The cytosolic glyoxalases of *Plasmodium falciparum* are dispensable during asexual blood-stage development

Cletus A. Wezena[1], Romy Alisch[1], Alexandra Golzmann[2], Linda Liedgens[1], Verena Staudacher[1,3], Gabriele Pradel[2] and Marcel Deponte[1,3,*]

Supplementary Materials and Methods

Table S1. Primers for cloning and analytical PCR used in this study. Restriction sites are underlined. The mutated start codon in P1 and the gRNA sequences in primers P9-P12 are italicized.

Primer #	Sequence
P1	5'-GATC*CCGCGG***ATT**GCACAAGAAATATCAAATTTAG-3'
P2	5'-GATC*TCTAGA*CCAATAATTATTAGGATCTAAAGC -3'
P3	5'-GATC*GAATTC*CAATGATTAGGGTTAAGAACCC-3'
P4	5'-GATC*CCATGG*TTTGCAATAAATGAAGTGTCCC-3'
P5	5'-GATC*CCGCGG*GTAGCCATGCGCACAAGTAC-3'
P6	5'-GATC*TCTAGA*TTGGTTCATATGCTGATCCGAC-3'
P7	5'-GATC*GAATTC*CGACGGTCAAATTATACGTTTAG-3'
P8	5'-GATC*CCATGG*TTAAGGGTATACTCATGTCCGC-3'
P9	5'-TAAGTATATAATATT***AAAGATCCAATCAAGTTAAA***GTTTTAGAGCTAGAA-3'
P10	5'-TTCTAGCTCTAAAAC***TTTAACTTGATTGGATCTTT***AATATTATATACTTA-3'
P11	5'-TAAGTATATAATATT***TACTTTCTCGTTTACACCTG***GTTTTAGAGCTAGAA-3'
P12	5'-TTCTAGCTCTAAAAC***CAGGTGTAAACGAGAAAGTA***AATATTATATACTTA-3'
P13	5'-CTCTTGGAGAACTCGCTGATCTG-3'
P14	5'-ACCGAGATTACCCTGGCCAACG-3'
P15	5'-GTGAGTATAGATCCTCATAACAACTTACG-3'
P16	5'-CATTGAATAATTGTGCATATATGATATACACAC-3'
P17	5'-CCTTTCTCCTCCTGGACATC-3'
P18	5'-CATGGTTCGCTAAACTGCATC-3'
P19	5'-CGAGGATATGATTTCTTTATTTTTATAACC-3'
P20	5'-AATCCTTATAAACAGTGACACATTACAC-3'

Figure S1

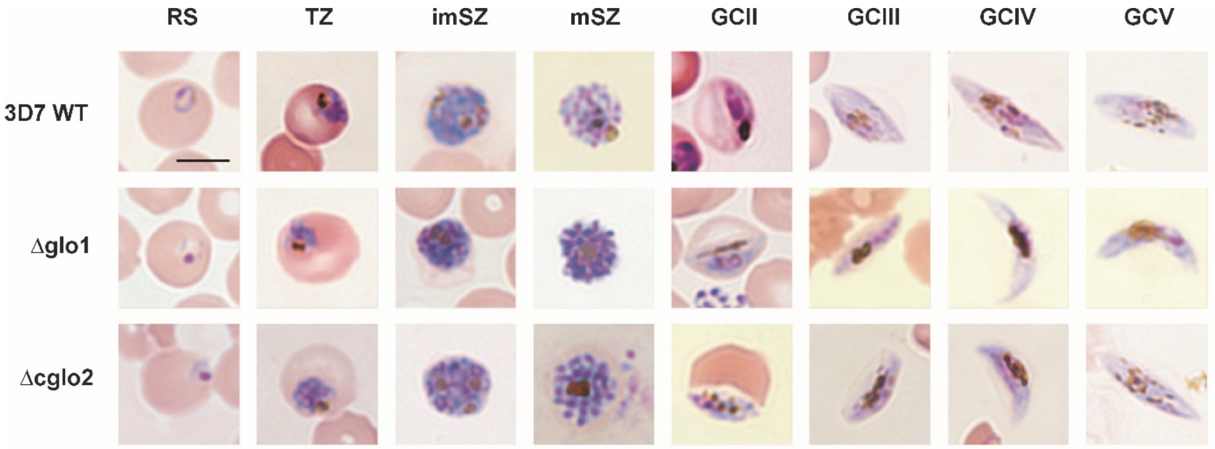

Fig. S1. Morphology of the 3D7Δglo1 and 3D7Δcglo2 asexual blood and gametocyte stages. Giemsa smears of ring stages (RS), trophozoites (TZ) and immature (imSZ) and mature (mSZ) schizonts as well as of gametocytes (GC) of stages II-V were microscopically analyzed and compared to the wild-type 3D7 blood stage parasites. Bar, 5 µm.

Figure S2

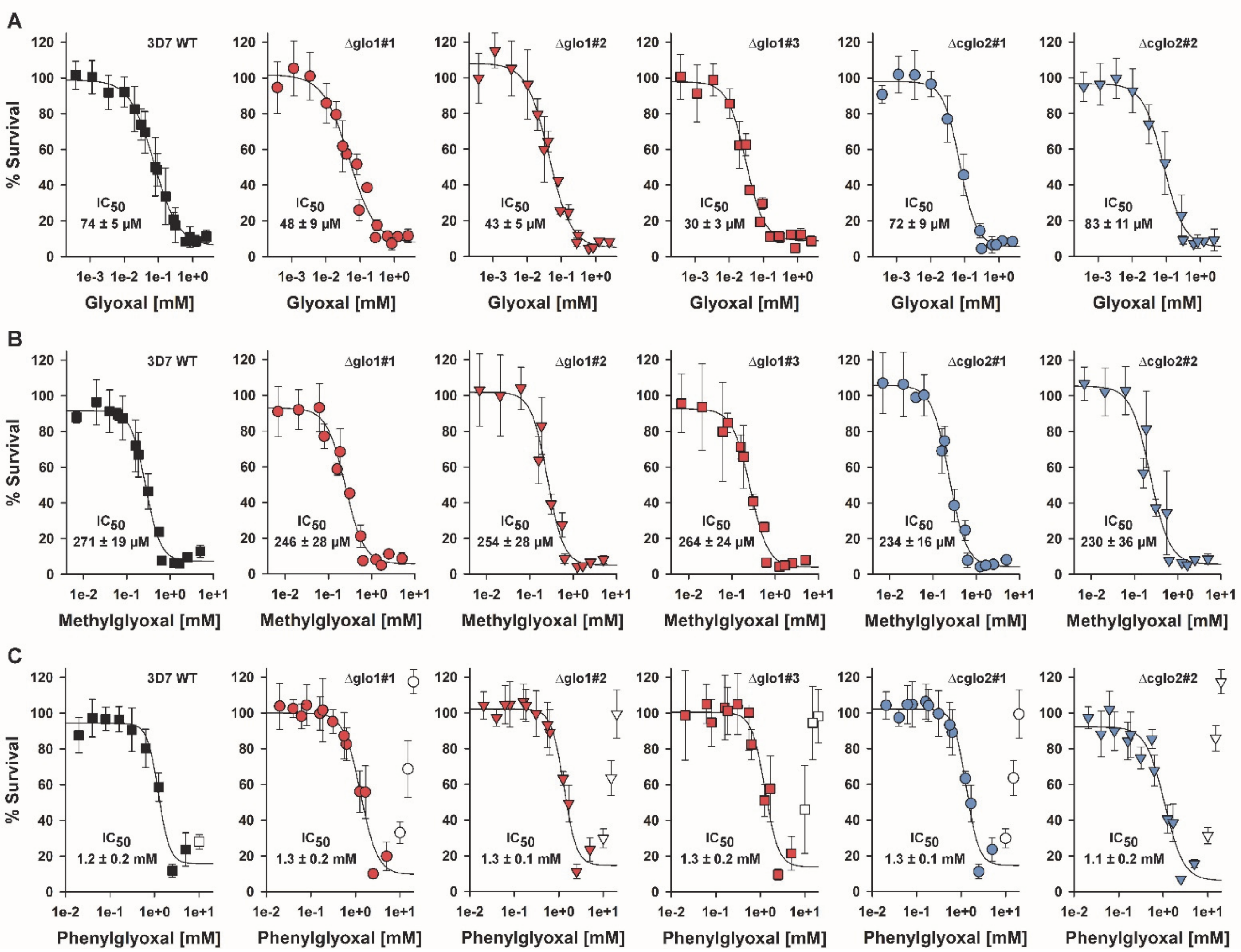

Fig. S2. Growth inhibitory effects of exogenous 2-oxoaldehydes on synchronous ring-stage cell cultures of *P. falciparum* wild-type strain 3D7 and clonal 3D7Δglo1 and 3D7Δcglo2 knockout strains. The initial hematocrit and parasitemia were 1.5% and 0.5%, respectively. IC_{50} values were determined in 96-well plates using a SYBR green 1 assay for bolus treatments with (A) glyoxal, (B) methylglyoxal and (C) phenylglyoxal after 72 h incubation. All data points are the mean ± S.D. from technical triplicate measurements of three to seven independent experiments. Data points at phenylglyoxal concentrations ≥10 mM were omitted because of a strong autofluorescence. The IC_{50} values are summarized in Fig. 4.
